# Detection of Remaining Feed in the Feed Troughs of Flat-Fed Meat Ducks Based on the RGB-D Sensor and YOLO V8

**DOI:** 10.3390/ani15101440

**Published:** 2025-05-16

**Authors:** Xueliang Tan, Junjie Yuan, Shijia Ying, Jizhang Wang

**Affiliations:** 1 School of Agricultural Engineering, Jiangsu University, Zhenjiang 212013, China; abc1049546807@126.com (X.T.); yuanjunjie@ujs.edu.cn (J.Y.); 2 Institute of Animal Science, Jiangsu Academy of Agricultural Sciences, Nanjing 210014, China; ysj@jaas.ac.cn

**Keywords:** localization of feed troughs, deep learning, point cloud processing, volumetric determination

## Abstract

The remaining feed in the feed troughs affects the feeding management of flat-raised meat ducks. Ground-contact detection methods all involve modifications to the feeding troughs, but the breeding setting of flat-raised meat ducks does not allow for on-site electrical wiring installation. Additionally, the existing non-contact methods do not directly detect the remaining feed quantity in the feeding troughs. To tackle this problem, this study employs a novel approach by first capturing images of the feed troughs using an RGB-D sensor. Subsequently, YOLOv8 is utilized to identify the positions of the feed troughs, and the volume of the remaining feed is determined through point cloud processing. The accuracy of this detection method was evaluated using various types of feed troughs and feed particle sizes. The experimental results reveal both a strong correlation between the calculated and actual feed volumes (with R^2^ values exceeding 0.86, indicating a consistent trend) and a low prediction error, as quantified by the root mean square error (RMSE). Analyses of the correction coefficients and corresponding RMSE values indicated a positive correlation between the correction coefficient and the curvature of the feeding trough, while no correlation was observed with the trough diameter or granule particle size, maintaining a low RMSE value. The findings of this research demonstrate the effectiveness of the proposed method for detecting the remaining feed in troughs. This method facilitates precise feed management, minimizes residual feed, and enhances the living conditions of meat ducks.

## Simple Summary

Accurately measuring the remaining feed in duck troughs is crucial for animal health and waste reduction, but the current methods are rendered ineffective due to the inability to install electrical wiring in the environment of flat-raised meat duck husbandry. To address this, the researchers created a system using a 3D camera to photograph feed troughs. It locates troughs with image analysis and calculates the remaining feed volume using depth data. Tests with different trough shapes and feed particle sizes showed that the system’s estimates closely matched real volumes, achieving over 86% accuracy. The method performed best with curved troughs but worked consistently regardless of trough size or feed type. This technology helps farmers precisely monitor feed levels, minimizing waste and ensuring cleaner environments for ducks. By improving feed management, it supports sustainable farming, reduces costs, and enhances animal welfare. This study highlights how practical technological solutions can tackle agricultural challenges, benefiting both farmers and animals.

## 1. Introduction

With the global economic recovery and rising demand for high-protein nutrition, meat ducks have emerged as an affordable and ideal protein source. The statistics from the Food and Agriculture Organization (FAO) of the United Nations (2018–2023) [1] indicate a steady annual growth in global duck meat production, with Asia accounting for 89.6% of the total output and China leading worldwide production. In modern meat duck husbandry, excessive feed distribution poses dual challenges: substantial feed waste and increased bacterial proliferation in high-temperature, high-humidity environments, both detrimental to animal welfare and operational efficiency.

The global research on material pile detection offers valuable insights. Tian [2] developed a heterogeneous camera system integrating enhanced DeepLabV3+ monocular detection and CREStereo binocular networks, achieving 92.9% accuracy in volumetric measurements of coal and aggregate piles. Similarly, Vacca [3] utilized UAV photogrammetry with Metashape Professional 1.7.2 and Cloud Compare software (official website: https://www.cloudcompare.org/, accessed on 12 May 2025) for sand quarry volume analysis, while Liu [4] proposed a monocular vision system achieving 93.06% peak-state accuracy in concrete aggregate measurement. Dahl [5] demonstrated via radar simulation that for a silo with a diameter of 10 m and height of 60 m, using an antenna beam width of 5° and bulk material penetration depth of 2 m, the surface height measurement error remained below 2 m under a minimum signal-to-noise ratio (SNR) of 10 dB. Despite these successes in flat-surface pile measurements, their direct application to meat duck husbandry is limited by the non-planar geometry of feed troughs.

The livestock industry has consequently focused on sensor technologies and trough design modifications for residual feed monitoring. Wang [6] developed an STM32-based capacitive sensor for bran thickness measurement in swine troughs, and Shuai [7] implemented a plate-voltage detection system for similar applications. Rumphorst [8] and Davison [9] employed automated weighing systems for cattle-feed-intake monitoring, paralleled by Gates’ [10] precision scale implementation in poultry operations. However, these contact-based methods prove unsuitable for open flat-fed duck environments where power line installation risks animal welfare and operational safety.

Non-contact measurement technologies leveraging optical and electromagnetic principles have emerged as promising alternatives. Advances in computer vision—particularly deep learning algorithms—have revolutionized object recognition, though volumetric analysis requires depth integration. Hu [11] proposed *Point2PartVolume,* a method predicting body volumes from single dressed-body depth images with 90% accuracy on synthetic and real-world datasets. Lo [12] developed *Point2Volume,* achieving 95.16% training and 92.29% testing accuracies in food volume estimation. In livestock applications, deep learning-driven measurement approaches have demonstrated significant potential: Bezen [13] improved cattle-feed-intake measurements using CNNs with RGB-D cameras, while Saar [14] achieved MAE/RMSE values of 0.14/0.19 kg/meal in bovine feed-prediction models. Shelley [15] validated 3D volume scanning for feed weight estimation with 0.5 kg accuracy, and Peng [16] developed a non-contact, automated cattle measurement system with improved keypoint detection and unilateral depth imaging, achieving 1.28–6.47% mean relative errors in 23 beef cattle tests, highlighting robustness in livestock precision measurements.

Technological progress in point cloud-based volume measurements has followed a structured evolution. Early LiDAR-focused studies, such as Molina’s [17], identified the limitations of traditional GPS (low sampling density, manual inspection risks) and demonstrated that UAS-mounted LiDAR systems outperform GPS in efficiency and accuracy. Cui [18] advanced this by integrating LiDAR with deep learning for bulk pile segmentation, achieving a <2% relative error via triangular meshing and Bessel surface fitting. However, LiDAR’s inability to utilize color information and a lower resolution compared to RGB-D cameras spurred the exploration of alternative sensors.

Complementary stereo camera approaches emerged next: Yin [19] developed a ZED camera-based method using spatial scaling, achieving a 7% average error with a robust real-time performance. Guevara [20] enhanced this by segmenting loader buckets from 3D point clouds via machine learning, achieving 95% accuracy in ZED stereo camera tests. Concurrently, 2D-3D fusion techniques gained traction: Bellandi [21] validated combining color image features with 3D instance segmentation for stockpile analysis, while Ni [22] and Sari [23] extended this to agriculture—Ni achieving 97.3% fruit counting accuracy via Mask R-CNN and photogrammetry, and Sari achieving ≤3.1% error in food volume estimation using K-means clustering and ellipsoid fitting.

Later research focused on environmental adaptability and application specificity. Rogers [24] paired LiDAR with a stereo camera to address dust/lighting challenges, demonstrating the camera’s dust-penetration capability in grain volume measurement and real-time vessel loading. Tuan [25] optimized stereo vision for construction slump measurement, achieving ≤2.05% geometric error and <8.9% volume error at 1.7–1.9 m working heights with a 70 mm baseline. In agriculture, Moreno [26] leveraged the low-cost Kinect v2 for vineyard monitoring, establishing strong correlations between sensor-derived branch volume and pruning weight (R^2^ = 0.80) or yield (R^2^ = 0.87), highlighting its potential for non-destructive biomass assessment.

The existing studies predominantly focus on feeder structural improvements, yet flat-fed meat duck systems require non-contact detection to eliminate wire-related tripping and stampede risks. Critical limitations persist in the current non-contact methods: unmodeled irregular feed accumulation in troughs and geometric disparities between circular (closed-boundary) and toroidal (hollow-center) trough contours reduce cross-feeder adaptability; dynamic lighting degrades point cloud RGB features, while 2D image-based approaches—despite gray-scale normalization to mitigate light interference—lack depth data, causing substantial volume errors during feed height variations from duck foraging (e.g., spreading/piling). Among non-contact solutions, RGB-D sensors stand out for their cost-effectiveness and accuracy, as shown in prior trough volume studies. This research innovatively applies RGB-D technology to flat-fed meat duck husbandry, introducing a novel framework for residual feed volume measurement. By enabling real-time quantity monitoring, our approach facilitates precise feed management, reducing waste and optimizing feeding schedules. This study’s originality lies in adapting computer vision techniques to open poultry environments, addressing industry challenges in resource efficiency and animal welfare enhancement.

## 2. Materials and Methods

### 2.1. Data Source

#### 2.1.1. Experimental Materials

This experiment was conducted in a laboratory located in Zhenjiang City, Jiangsu Province, China, focusing on feed materials commonly used in meat duck husbandry. To investigate the influence of feed physical properties on the accuracy of residual feed volume detection, four types of feed were selected: CZ small-duck compound feed (548), CZ medium/large-duck compound feed (549L), egg-laying-duck compound feed (544), and large-duck feed. These feeds exhibited distinct variations in particle size and length, providing a representative sample for analysis. The physical properties of each feed type—length, diameter, mass, and density—were quantitatively evaluated using precision instruments: a digital vernier caliper (accuracy: 0.01 mm) for measuring particle length and diameter, and an electronic scale (precision: 0.01 g, capacity: 500 g) for determining mass (density was calculated from mass and volume measurements). Each property was measured across 10 representative samples per feed type, with mean values and standard deviations for the four properties summarized in Table 1, enabling the statistical characterization of inter-sample variability. Detailed measurement results are summarized in Table 1.

In order to understand the effect of the diameter and category of feed troughs on the detection method of the RGB-D sensor, three feed troughs with different diameters of the same category and another two different categories were assessed in this study, which were plastic-barrel snap handles with capacities of 3 kg and 4 kg, iron-barrel snap handle with a capacity of 10 kg, a 2 kg feed trough, and a 25 cm green, plastic feed trough.

This study employed the Azure Kinect depth camera, a widely used RGB-D sensor [27], to detect feed troughs and measure the residual feed volume in meat duck husbandry settings. The camera was positioned 1.96 m above the troughs and utilized the Time-of-Flight (TOF) principle for depth sensing. Operational parameters included a frame rate of 30 fps, BGRA32 color mode at a 2160p resolution, and NFOV_UNBINNED depth mode, with the infrared functionality disabled to ensure consistent performance. In the experimental setup, the detection area of the depth camera [28] was determined by its depth module. When installed at 1.96 m, the camera’s field of view covered a ground area of approximately 2.598 m^2^. To align with the standardized layout of feed troughs in actual production—where adjacent troughs are spaced 2.5 m apart from center to center—the experimental design ensured that each depth camera was configured to monitor only one feed trough, maintaining consistency with real-world operational arrangements.

#### 2.1.2. Platform Description

For data processing, Visual Studio was employed, integrating essential libraries, including PCL 1.12.0, Azure Kinect Viewer v1.4.1, OpenCV 4.4.0, and VTK 9.0.2. This setup was selected to align with the C++ programming environment of the Azure Kinect depth camera, ensuring compatibility and efficiency. Consistent device configurations were maintained throughout data acquisition and deep learning processes.

The YOLOv8 model was implemented using the PyTorch 2.5.0 framework. Experiments were conducted on a Windows 11 64-bit system equipped with a 13th Gen Intel^®^ Core™ i9-13900HX processor and an NVIDIA GeForce RTX 4060 GPU. The software environment included CUDA 11.8, CUDNN 9.5.0, Labelme 3.16.2, PyCharm Community Edition 2024.2.3, and Python 3.10, providing a robust platform for model training and evaluation.

In data processing, Excel was used for linear regression on 12 training datasets, deriving R^2^ and the regression equation. RMSE evaluations were conducted for three groups of prediction datasets. RMSE is reported as two metrics: Train RMSE (model fit on training data) and Predict RMSE (prediction accuracy on new data), presented separately to evaluate model stability and generalization.

### 2.2. Detection of Remaining Feed

**Figure 1:**
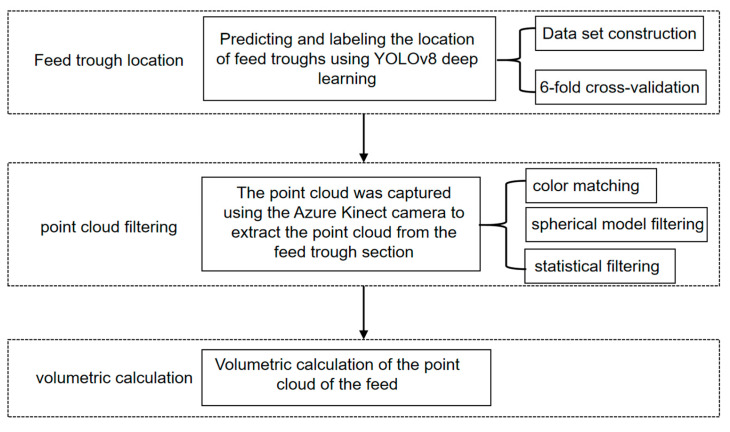
Flowchart for detecting the volume of the remaining feed in feed troughs by the RGB-D sensor.

To address the interference of ambient light on point cloud processing, we integrated the YOLOv8 model with point cloud filtering techniques for the accurate estimation of residual feed volume in the feed troughs. The proposed methodology was implemented in three sequential steps, as illustrated in Figure 1.

The methodology consisted of three main steps for detecting the volume of remaining feed in a meat duck husbandry setting:

(1) The feed trough location was determined using the Azure Kinect’s color-image module. A comprehensive feed trough dataset was constructed and trained using the YOLOv8 model, incorporating 6-fold cross-validation to ensure optimal model selection and performance validation.
(2) Point cloud processing was performed through multiple filtering stages. The depth camera of Azure Kinect aligned the color images with raw depth point cloud data to determine the feed troughs’ center-of-mass coordinates. A spherical model filtering approach was then applied for feed trough segmentation, followed by statistical filtering to eliminate outlier points. The unification of the coordinate system was achieved by aligning the center of the processed point cloud’s bounding box with the coordinate origin.
(3) Feed volume calculation was achieved by comparing the point cloud data of the feed trough containing feed with corresponding reference models from a pre-established trough library. This comparison enabled the isolation of feed-specific point cloud data, from which the precise volume of feed was calculated.

### 2.3. Feed Trough Location

This study collected color images of feed troughs at varying time intervals to construct a comprehensive dataset. Following dataset annotation and the application of data augmentation techniques, 6-fold cross-validation was implemented with the YOLOv8 model to identify the optimal model configuration. This approach ensured the accurate localization of feed troughs, which served as a critical input for subsequent point cloud processing.

#### 2.3.1. Dataset Construction

Color images (*n* = 345) of the feed troughs with the remaining feed were captured at 10 s intervals using an Azure Kinect depth camera at a laboratory in Zhenjiang City, Jiangsu Province. The images were saved in a JPG format with a resolution of 3840 × 2160 pixels.

#### 2.3.2. Data Annotation

Based on the characteristics of the feed trough, this study utilized Labelme 3.16.2 software to annotate color images with circular bounding boxes, storing the annotation results in a JSON format. The JSON files were processed to extract both the circle centers and circumference point coordinates. These circular annotations were then converted to square bounding boxes, with the circle’s center serving as the square’s center of mass and the circle’s diameter determining the square’s side length. To comply with deep learning requirements, the annotation format was modified from circular to rectangular specifications. Finally, the processed annotation files were organized into appropriate training folders.

#### 2.3.3. Classification of the Dataset

In this study, first, 345 photos were divided into a training set (276 photos) and a test set (69 photos) at a ratio of 4:1. Subsequently, a 6-fold cross-validation method was adopted. The training set (276 photos) was divided into a training subset (230 photos) and a validation subset (46 photos). Data augmentation was performed on the training subset (230 photos) for each model. Through methods such as random cropping, flipping, scaling, and color adjustment, the number of the training subset was increased to 10 times, that is, the size of the training set for each model reached 2300 photos. Next, model training, validation, and testing were carried out. A total of 6 rounds were conducted, and different validation subsets were used for validation in each round. Finally, six different models were obtained through training.

#### 2.3.4. YOLOv8 Target Detection

YOLOv8 [29], introduced in 2023, represents an advanced neural network architecture that enhances its predecessor, YOLOv5, to address multiple computer vision tasks, including image classification, object detection, and instance segmentation. The model is available in five variants (n, s, m, l, and x), each offering distinct trade-offs between computational complexity and performance. For this study, YOLOv8s was selected as the optimal configuration, balancing detection accuracy and processing efficiency given the dataset’s modest size.

#### 2.3.5. YOLOv8 Training Parameters and Evaluation Indicators

The 6-fold cross-validation experiments were conducted under identical system configurations, with the YOLOv8 neural network training parameters detailed in Table 2. To identify the optimal model, comprehensive evaluations were performed on all six trained models. Performance was assessed using four key metrics: precision, recall, mAP_0.5,_ and mAP_0.5–0.95_, with the results summarized in Table 3. Notably, all four metrics achieved an exceptional performance. Given the strict requirements for positioning accuracy and the dimensional accuracy of detection boxes in feeding-trough location detection, and considering the overall performance of object detection models across multiple IoU thresholds, after a comparative analysis of each model’s mAP_0.5–0.95_ index, Model 5, which demonstrates the best performance in this key metric (mAP_0.5–0.95_ = 0.887), was selected as the final prediction model for feeding-trough object detection.

### 2.4. Point Cloud Filtering

**Figure 2:**
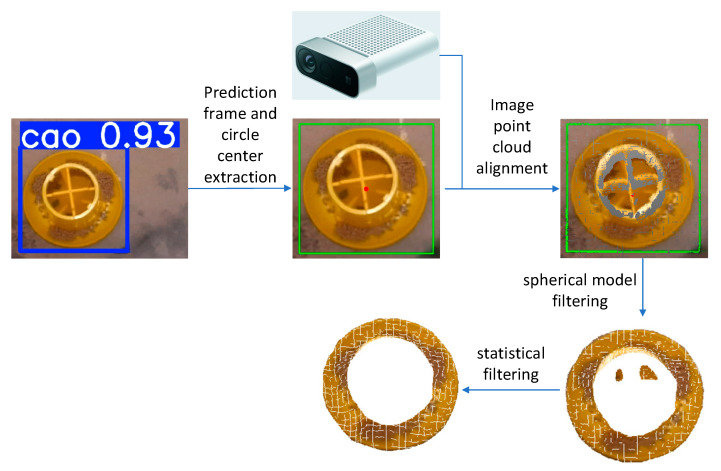
Point cloud data processing workflow, The red point in the figure is the centroid of the green prediction box.

After localizing the feed trough using YOLOv8, this study aligned the predicted color images with depth information from Azure Kinect. The centroid coordinates of the feed trough were extracted from the registered colored point cloud. Using the centroid as the sphere center, the feed trough’s point cloud data were isolated from the complex environment. Statistical filtering was then applied to remove outlier points, yielding the final point cloud data of the feed trough. The complete point cloud processing workflow is illustrated in Figure 2.

1. The optimal Model 5 was employed for prediction, generating images with prediction boxes. These boxes were extracted and annotated in green, followed by centroid extraction from the green prediction boxes, which were then marked in red.
2. The predicted color images were registered with the collected point cloud through the Azure Kinect camera, enabling the visualization of the processed green boxes and red centroids on the point cloud. Cloud Compare software was utilized to examine the point cloud coordinates and locate the corresponding coordinates of the red centroids.
3. Given the characteristics of feed troughs and the complex duck husbandry setting, this study implemented a sphere model filtering approach to segment the feed trough point cloud from the environmental point cloud. The process began by using the previously obtained centroid coordinates as sphere centers. Appropriate parameters were then selected from a pre-established feed trough model database, and the feed trough point cloud was segmented using sphere model filtering. The feed trough model database contains five preset models with corresponding parameters. For the 3 kg and 4 kg plastic-barrel snap handles, the sphere centers are located at the top of the feed trough point cloud. For the 10 kg iron-barrel snap handle, the sphere center is positioned 0.09 m above ground level. The 2 kg feed trough’s sphere center is situated 0.045 m above ground level, while the 25 cm green, plastic feed trough’s sphere center is located at the bottom center. Unlike the 25 cm green, plastic feed trough, which accommodates feed throughout its entire volume, other troughs only allow feed placement in their circular peripheral regions. To address this, threshold judgments were incorporated into the sphere model, retaining only point clouds within the defined threshold regions. The sphere model radii and threshold parameters are detailed in Table 4. Notably, the diameter values refer to the actual bottom diameter of the feed trough, not the sphere model diameter. For the 25 cm, green plastic feed trough, the sphere model diameter was calculated as (0.073 + 0.073) × 2 = 0.292 m. For other troughs, the threshold was set to half the actual circular ring width, with radii derived using the Pythagorean theorem based on sphere center height and the actual radius minus the threshold.
4. The segmented point cloud still contained outliers, which were removed using statistical outlier removal filtering—a method that eliminates points with abnormal neighborhood distances based on Gaussian distribution statistics. Considering the point cloud density and filtering effectiveness, this study set the number of neighboring points (k-nearest neighbors, k = 1000) and the standard deviation multiplier (σ-multiplier = 1.0), ensuring high-quality point cloud data for subsequent processing.
5. According to Fernández-Sarría [30], among the five methods, including convex hull, convex hull by slices of 5 cm height in the XY plane, triangulation by XY flat sections, and voxel modeling, the voxel modeling method exhibited the highest goodness of fit, with an R^2^ value of 0.731 for the relationship between crown volume and residual biomass. Therefore, the method used in this paper to handle volume was voxelized modeling. Considering the spatial correspondence (i.e., the degree of one-to-one structural alignment) between the upper and lower layers of voxels and the need for adequate voxel density to capture fine spatial details, voxels with a size of 2 mm × 2 mm × 2 mm were selected for volume calculation. However, voxelized volume calculation requires that the two point clouds be unified in the same coordinate system. The solution adopted in this study is to extract the bounding box of the point cloud after statistical filtering and align the center of the bounding box to the origin.

### 2.5. Volumetric Calculation

**Figure 3:**
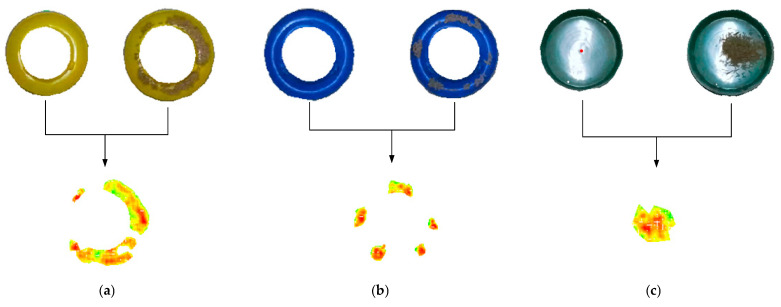
The process of detecting the volume of the remaining feed. The height of the feed is quantified in the figure, where the darker the color, the greater the height difference; the red dot in the figure is the centroid of the prediction box. (**a**) The processing procedure of the plastic-barrel snap handle with a capacity of 3 kg; (**b**) the processing procedure of the 2 kg feed trough; (**c**) the processing procedure of the 25 cm green, plastic feed trough.

**Figure 4:**
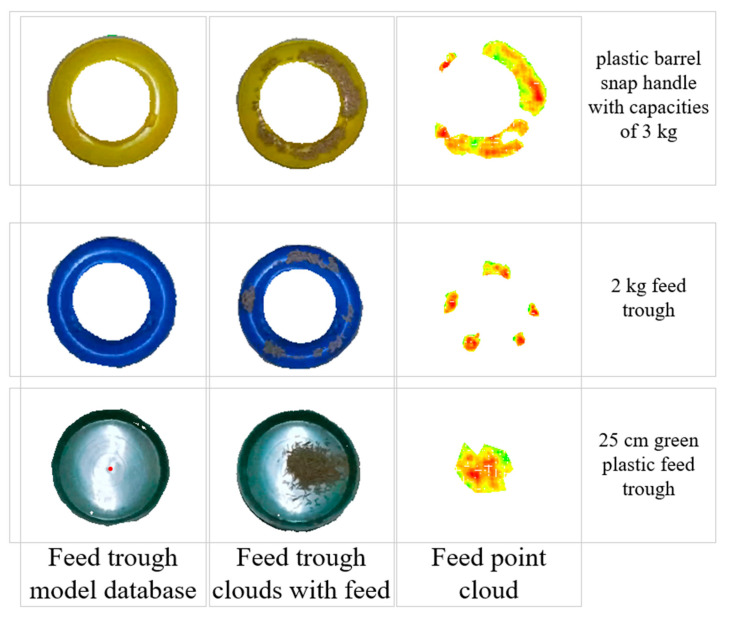
The process involves vertically overlapping the model library’s empty feeding trough with the feed-filled one to select the height-differential point cloud (i.e., the feed region). The figure illustrates image sources, quantifies feed height with darker shades indicating greater differences, and marks the prediction box centroid with a red dot.

This study focuses on detecting the volume of residual feed in troughs using point cloud data. Since point clouds only capture surface depth information and cannot directly measure feed depth within the trough, a specialized extraction method was developed. The approach involved merging point cloud data from filled troughs with corresponding empty trough models from the feed trough model database. Points with height differences below a predefined threshold were removed to isolate the feed portion. Based on the depth differences derived from the merged point clouds, the feed volume was calculated. Figure 3 illustrates the process of extracting feed point clouds for three types of feeding troughs, while Figure 4 mainly shows the source of each point cloud during the feed point cloud processing procedure.

## 3. Results

### 3.1. The Positioning Result of the Feed Troughs

**Figure 5:**
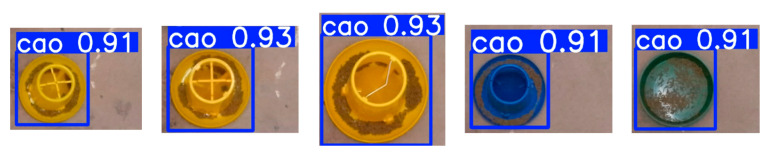
Results of feed trough detection.

The training results of YOLOv8 are shown in Figure 5. This figure presents the detection results of five types of feed troughs. It can be seen that the trained model demonstrates a robust performance in accurately identifying and localizing the target feed troughs.

### 3.2. Detection Results for Different Types of Feeding Troughs

In this study, large-duck feed was used as the test material to analyze the feed volume in three types of feeding troughs, each with a diameter of 250 mm. Building on the methodology [31] of using Kinect v1 to measure the grape cluster volume with correction processing, this paper introduces a correction coefficient defined as the ratio of the actual volume to calculated volume. The actual volume was determined by first measuring the mass of feed in the trough and then calculating the true volume through the established mass–volume relationship, a critical step that anchors geometrically derived raw volume data to real-world mass measurements and mitigates errors from material density variations or trough geometry inconsistencies. This coefficient is applied to calibrate and optimize the data collected by Azure Kinect, extending the correction framework to the new sensor system.

To validate the correction strategy, the coefficient of determination (R^2^) and root mean square error (RMSE) are used: R^2^ evaluates the regression model’s explanatory power for adjusted volumes, while RMSE quantifies the average discrepancy between calculated and true values, together ensuring a concise assessment of the correction’s effectiveness.

**Figure 6:**
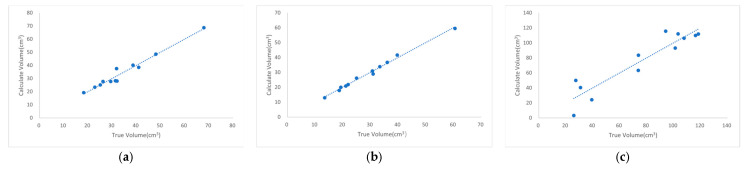
Regression curves of calculated volume (as the *y*-axis) versus actual volume (as the *x*-axis) for different feed trough categories using the same type of feed—data points represent experimental measurements of both values, and regression lines model their relationship: (**a**) plastic-barrel snap handle with a capacity of 3 kg; (**b**) 2 kg feed trough; (**c**) 25 cm green, plastic feed trough.

Figure 6 shows the regression curves between the calculated volume and true volume of different types of feeding troughs using this method, while Table 5 presents the data extraction and explanation of the predicted RMSE in the table. Among the three trough types, the 3 kg plastic-barrel snap handle exhibited the largest correction coefficient, followed by the 2 kg feed trough, while the 25 cm green, plastic feed trough had the smallest value. The R^2^ values for all three trough types exceeded 0.86, indicating a strong linear fit. In terms of Train RMSE, the values were 2.46 cm^3^ for the 3 kg plastic-barrel snap handle, 1.03 cm^3^ for the 2 kg feed trough, and 13.65 cm^3^ for the 25 cm green, plastic feed trough. The RMSE trends of the prediction datasets are consistent with those of the training datasets, where the largest, middle, and smallest RMSE values in the prediction datasets respectively correspond to those in the training datasets.

### 3.3. Detection Results of Feeding Troughs with Different Diameters

**Figure 7:**
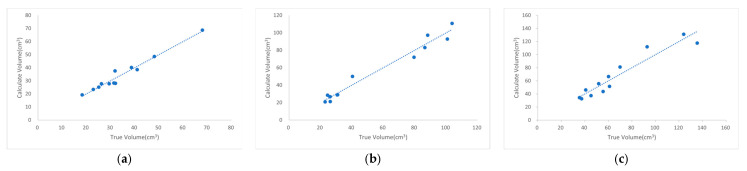
Regression curves of calculated volume (*y*-axis) versus actual volume (*x*-axis) for feed troughs of different diameters when using the same type of feed—data points represent experimental measurements of both values, and regression lines model their relationship: (**a**) Feed trough with a diameter of 0.25 m; (**b**) feed trough with a diameter of 0.27 m; (**c**) feed trough with a diameter of 0.38 m.

In this study, the volume of large-duck feed was calculated for troughs with three different diameters. Figure 7 illustrates the regression curves between the calculated and true volumes of feeding troughs with different diameters using this method, whereas Table 6 provides details of data extraction and explanations for the predicted RMSE. The R^2^ values between the calculated and actual volume values exceeded 0.90, indicating a strong linear fit. Regarding the correction coefficient, the values for the two troughs with similar diameters were close, while the coefficient for the larger-diameter trough was significantly higher. In terms of Train RMSE, the values showed a positive correlation with trough diameter. Specifically, the 3 kg plastic-barrel snap handle (diameter: 0.25 m) had the lowest Train RMSE, the 4 kg plastic-barrel snap handle (diameter: 0.27 m) ranked in the middle, and the 10 kg iron-barrel snap handle (diameter: 0.38 m) exhibited the highest Train RMSE. The trends of Predict RMSE were consistent with those of Train RMSE, showing a one-to-one correspondence.

### 3.4. Detection Results of Feed Particles with Different Particle Sizes

**Figure 8:**
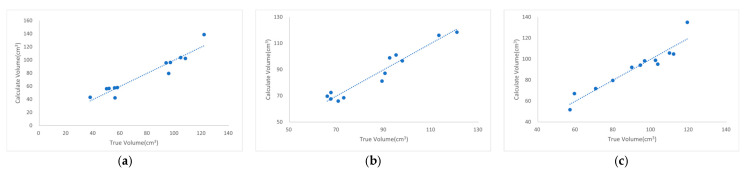
Regression curves of calculated volume (*y*-axis) versus actual volume (*x*-axis) for feed particles of different sizes when using the same feed trough—data points represent experimental measurements of both values, and regression lines model their relationship: (**a**) CZ small-duck compound feed (548); (**b**) CZ medium/large-duck compound feed (549L); (**c**) egg-laying-duck compound feed (544).

Three feed types with distinct particle sizes were analyzed: CZ small-duck compound feed (548), CZ medium/large-duck compound feed (549L), and egg-laying-duck compound feed (544). A 25 cm green, plastic feed trough with a flat bottom was used as the measurement container. Figure 8 illustrates the regression curves between the calculated and true volumes of feed particles with different sizes using the same feed trough, whereas Table 7 provides details of data extraction and explanations for the predicted RMSE. For all three feed types, the coefficient of determination (R^2^) for the relationship between calculated and actual volume values exceeded 0.90, demonstrating a strong linear correlation. Importantly, the correction coefficients, Train RMSE, and Predict RMSE values remained highly consistent across the feed types. This indicates that variations in feed particle size exerted a minimal impact on the detection method’s performance, whether evaluated on the training datasets (Train RMSE) or the prediction datasets (Predict RMSE).

## 4. Discussion

Building on the demonstrated potential of RGB-D sensors for agricultural volume monitoring, this study utilizes the Azure Kinect camera [26], which provides superior point cloud accuracy and enhanced color performance compared to the ZED camera [19,20] and D435i [23], to advance non-destructive crop structure analysis. Previous research has demonstrated the potential of depth cameras in agricultural applications, such as estimating corn volume [24] and performing instance segmentation on both 2D color images and 3D point clouds [21]. Leveraging these capabilities, we utilized the Azure Kinect camera to capture color images for the precise localization of the feed trough. Subsequently, the depth module of the Azure Kinect was employed to conduct instance segmentation on the processed point cloud of the feed trough, enabling the extraction of the point cloud representing the remaining feed. The volume of this extracted point cloud was then calculated. Supporting our methodology, the literature [25] proposed stereo cameras for image capture and depth map integration to calculate slump, while complementary studies, like Ni et al.’s [22] (2D instance segmentation + 3D photogrammetry for blueberry clusters) and Sari et al.’s [23] (2D RGB segmentation + 3D point cloud fitting for food volume), demonstrate multi-dimensional data fusion’s effectiveness, further validating our approach. Notably, these studies rely on model-fitting methods for volume calculation, which inherently lose structural details due to geometric assumptions. Such approaches are inadequate for feed volume calculation, due to the porosity of feed particles and the irregular shapes formed during feed accumulation, which necessitate detail-preserving methods beyond simplistic geometric fitting. Voxel-based methods, however, can effectively address this limitation by preserving structural details through discrete 3D grid representation [30].

However, several factors—such as the distance between the Azure Kinect depth camera and the feed trough [25], the resolution of the point cloud, the filling rate of feed particles, and the inherent accuracy of the camera [20]—interact in complex ways, leading to discrepancies between the detected volume and the actual volume. These interactions introduce errors that must be carefully considered in the analysis.

To address these discrepancies, we introduced a correction coefficient to adjust the detected volume values, which are subsequently compared with actual measurements. While references [24,25] focused on single-time data analysis, limiting their universality, our approach employs root mean square error (RMSE) for comprehensive error analysis. The reliability of our method is evidenced by an R^2^ value exceeding 0.86, demonstrating a strong linear relationship between calculated and actual volumes, while the low RMSE values indicate minimal deviation between measured and actual volumes. When the correction coefficient exceeds 1, it signifies that the actual volume is greater than the calculated volume. This discrepancy arises from differences between the physical model and its 3D point cloud representation, particularly in voxel-based calculations. The non-correspondence of upper and lower voxels reduces the number of calculable voxels, leading to an underestimation of the volume. Despite these advancements, our method has limitations. Notably, significant discrepancies persist between calculated and actual volumes for iron-barrel snap handles with capacities of 10 kg. Additionally, the current system lacks automated feed trough classification and relies on a manual parameter input for model selection, highlighting opportunities for further development toward full automation and intelligence. These limitations underscore the need for ongoing refinement to enhance the system’s accuracy and usability.

## 5. Conclusions

In this study, we developed a novel RGB-D sensor-based method to determine residual feed volume in meat duck husbandry settings, overcoming the limitations of traditional contact measurements and existing non-contact approaches. This methodology involves three sequential steps: feed trough localization via YOLOv8, alignment of color images with depth sensor point clouds, and volume calculation through spherical model fitting combined with the statistical filtering of processed point clouds.

Experimental validation across diverse trough types (varying diameters in the range of 25–38 cm) and feed particle sizes (2.2–4.8 mm) demonstrated a strong agreement between measured and actual volumes (R^2^ > 0.86). Critical analysis revealed that the correction coefficient correlates positively with trough bottom curvature, while showing no significant dependence on trough diameter or feed particle size. With a maximum RMSE of 25.62 cm^3^, this non-invasive system proves reliable for real-time feed monitoring in commercial duck farming operations.

## Figures and Tables

**Table 1:** Physical properties of feeds.

Feed Type	Length (mm)	Diameter (mm)	Mass (g)	Density (kg/m^3^)
548	5.95 ± 3.36	2.20 ± 0.06	0.05 ± 0.02	1164.22 ± 231.88
549L	7.42 ± 1.43	3.63 ± 0.11	0.08 ± 0.02	1118.47 ± 176.57
544	10.36 ± 1.38	4.83 ± 0.10	0.23 ± 0.04	1193.78 ± 89.23
Large-duck feed	15.08 ± 4.53	4.24 ± 0.20	0.25 ± 0.09	1167.68 ± 110.15

**Table 2:** Training parameters.

Parameter	Numeric Value
Training batches	6
Iterations	100
Image size	640 × 640
Initial learning rate	0.01
Momentum of learning step	0.937

**Table 3:** Training results for the 6-fold cross-validation model.

Model Number	Precision	Recall	mAP_0.5_	mAP_0.5−0.95_
1 *	0.980	0.982	0.987	0.871
2 *	0.976	0.959	0.983	0.871
3 *	0.983	0.982	0.985	0.866
4 *	0.951	0.960	0.980	0.862
5 *	0.979	0.972	0.989	0.887
6 *	0.972	0.956	0.984	0.856

* Precision and recall used YOLOv8 defaults: conf = 0.25, NMS IoU = 0.70, fixed across 6 folds.

**Table 4:** Parameters in the preset model library.

Feed Trough Type	Diameter (m)	Height (m)	Sphere Model Radius (m)	Sphere Model Threshold (m)
Plastic-barrel snap handle with a capacity of 3 kg	0.25	0.125	0.162	0.025
Plastic-barrel snap handle with a capacity of 4 kg	0.27	0.180	0.215	0.028
Iron-barrel snap handle with a capacity of 10 kg	0.38	0.295	0.175	0.040
2 kg feed trough	0.25	0.165	0.110	0.025
25 cm green, plastic feed trough	0.25	0.055	0.073	0.073

**Table 5:** Partial parameters of the regression curves of different types of feeding troughs.

Feed Trough Type	Correction Coefficient	R^2^	Train RMSE (cm^3^)	Predict RMSE (cm^3^)
Plastic-barrel snap handle with a capacity of 3 kg	3.22	0.964	2.46	8.37
2 kg feed trough	2.56	0.993	1.03	3.47
25 cm green, plastic feed trough	1.74	0.866	13.65	16.53

**Table 6:** Partial parameters of the regression curves of feed troughs with different diameters.

The Diameter of the Feed Trough (m)	Correction Coefficient	R^2^	Train RMSE (cm^3^)	Predict RMSE (cm^3^)
0.25	3.22	0.964	2.46	8.37
0.27	2.10	0.968	5.82	17.18
0.38	4.17	0.906	10.24	25.62

**Table 7:** Partial parameters of the regression curves of feed particles with different particle sizes.

Categories of Feed Particles	Particle Diameter (mm)	Correction Coefficient	R^2^	Train RMSE (cm^3^)	Predict RMSE (cm^3^)
548	2.20	1.08	0.911	8.52	8.42
549L	3.63	1.77	0.927	4.55	11.97
544	4.83	1.43	0.903	6.43	11.63

## Data Availability

The original contributions presented in this study are included in the article. Further inquiries can be directed to the corresponding author.
